# Intérêt de l’algorithme rocuronium - sugammadex dans la laryngoscopie directe en suspension

**DOI:** 10.11604/pamj.2017.26.232.11244

**Published:** 2017-04-25

**Authors:** Sidi Driss El jaouhari, Mohamed Meziane, Redouane Ahtil, Mustapha Bensghir, Charki Haimeur

**Affiliations:** 1 Pole d’Anesthésie-Réanimation, Hôpital Militaire d’Instruction Mohamed V, Faculté de Médecine et de Pharmacie, Université Mohamed V, Rabat, Maroc

**Keywords:** Laryngoscopie direct en suspension, intubation, rocuronium, sugammadex

## Abstract

La laryngoscopie directe en suspension est un geste chirurgical diagnostique et/ou thérapeutique des lésions endo-laryngées. Sa gestion anesthésique est compliquée. Différentes techniques anesthésiques peuvent être proposées. Malgré les contraintes, les curares gardent tout leur intérêt. L’association rocuronium et sugammadex est envisageable, elle permet une inversion rapide du bloc neuromusculaire profond et par conséquent une réduction de la morbidité postopératoire. Nous rapportons un cas d’une laryngoscopie directe en suspension réalisée sous anesthésie générale dont l’utilisation de l’association rocuronium-sugammadex a permis une facilité du geste chirurgicale, une sécurité pour le patient et un confort pour l’anesthésiste.

## Introduction

La laryngoscopie directe en suspension (LDS) est un geste chirurgical qui permet de préciser l’extension, de réaliser un prélèvement ou biopsie et d’effectuer un geste thérapeutique d’une lésion endo-laryngée. C’est un geste diagnostique et / ou thérapeutique dont les contraintes sont importantes: permettre au chirurgien de réaliser son geste dans les meilleures conditions, tout en assurant la sécurité des patients dont l’état général et la fonction respiratoire peuvent être précaires [1]. La gestion anesthésique durant la laryngoscopie en suspension est compliquée du faite du partage de voies aériennes supérieures entre chirurgien et anesthésiste. Cette gestion va dépendre du siège et de la taille de la lésion à explorer [2]. Différentes techniques anesthésiques peuvent être proposées. L’utilisation des curares garde toute son importance malgré les contraintes (geste court, risque d’intubation ou de ventilation difficile..). L’intérêt du Sugammadex est de permettre de récolter les bénéfices des curares stéroïdiens en toute sécurité sans risque de curarisation résiduelle. Nous rapportons un cas d’utilisation de Sugammadex après une laryngoscopie directe en suspension réalisée sous anesthésie générale avec curares.

## Patient et observation

**Figure 1:**
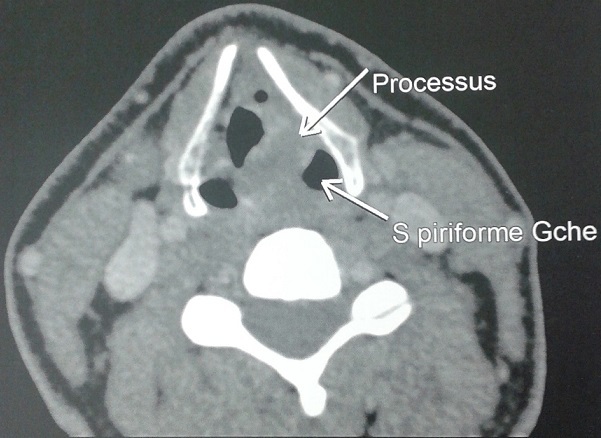
Image tomodensitométrique montrant le processus tumoral de l’hémilarynx gauche

Il s’agissait d’un patient de 60 ans, suivis depuis 10 ans pour broncho-pneumopathie chronique obstructive (BPCO), bien équilibrée sous traitement de fond à base de bronchodilatateurs et de corticoïdes inhalés. Opéré en 2013 pour exérèse d’une lésion de la base de la langue (cystadénolymphome). Dans ces antécédents toxiques on retrouvait un tabagisme chronique à raison de 42 paquets année, ainsi qu’un alcoolisme occasionnel. Le début de sa symptomatologie remontait à 4 mois par l’installation d’une dysphagie progressive à évolution intermittente sous traitement à base de corticoïdes, accompagné d’une dysphonie, le tout évoluant dans contexte d’altération de l’état général et d’amaigrissement chiffré à 12 kg en deux mois. L’examen clinique retrouvait un patient apyrétique, eupnéique au repos, sans adénopathies cervical palpable, le reste de l’examen somatique était sans particularités. La laryngoscopie indirecte avait objectivé un processus bourgeonnant de l’hémi larynx gauche et du sinus piriforme gauche. Une tomodensitométrie (TDM) du larynx retrouvait un gros processus tumoral manifestement malin de l’hémi larynx gauche (Figure 1), de 23,5 mm de diamètre transverse, 33 mm de diamètre antéropostérieur, et étendu en hauteur sur 31 mm, intéressant la corde vocale, le ventricule laryngé, le pli vestibulaire et comblant en partie le sinus piriforme gauche, ce processus lésionnel est responsable d’un phénomène de lyse osseuse de la paroi antérolatérale gauche du cartilage thyroïde et de l’aryténoïde gauche. Le patient était candidat d’une laryngoscopie directe en suspension.

L’évaluation pré-anesthésique retrouvait lors de l’examen de l’appareil respiratoire une dyspnée stade II de NYHA avec une tolérance modérée à l’effort. L’auscultation des deux champs pulmonaires retrouvait de faibles sibilants basaux bilatéraux, la pression non invasive (PNI) était de 141 mmHg/85 mmHg avec une fréquence cardiaque de 93 battements/min, la saturation pulsée en oxygène (SpO2) était de 95% à l’air ambiant. Le patient ne présentait pas de critères d’intubation ni de ventilation difficile décelable avec une bonne ouverture buccale et un Mallampati I ainsi qu’une distance thyromentonniére supérieur à 6cm. Les examens biologiques à savoir une numération de la formule sanguine, une fonction rénale, un bilan hépatique et radiologique préopératoires à savoir un ECG et une radiographie du thorax ne retrouvaient rien de particulier à part une polyglobulie expliquée par le tabagisme chronique. Au bloc opératoire, un monitorage classique contenant un enregistrement du rythme et de la fréquence cardiaque, la PNI, la Spo2 ainsi que la capnographie a été effectué. Une voie veineuse périphérique au membre supérieur droit a été prise et une pré-oxygénation à 100% de Fio2 a été entamée. L’anesthésie a été induite par du propofol 3mg/kg associé à la lidocaïne 1mg/kg, fentanyl 3ug/kg, et une injection du rocuronium à raison de 0,5mg/kg. Le patient a été intubé par un tube endotrachéal de 5,5 mm de diamètre et mis sous ventilation artificielle. 25 mg d’hydrocortisone a été administré. L’entretien de l’anesthésie a été assuré par sévoflurane 1,4% avec un mélange équimolaire oxygène / protoxyde d’azote. Le monitorage ventilatoire consistait en une surveillance rapprochée de la pression plateau avec comme objectif une pression inférieur à 30 cm H_2_O. Aucun incident per opératoire n’a été noté. A la fin du geste, et après arrêt des halogénés, une administration du sugammadex à raison de 4mg/kg a été effectué, la décurarisation était rapide et l’extubation a pu avoir lieu 5 minutes après avoir assuré une normothermie et une analgésie. Le geste a duré 15 minutes, et la durée totale d’anesthésie était de 25 minutes. Aucun incident n’a été noté en salle de surveillance post interventionnelle.

## Discussion

La LDS est une technique qui permet l’étude à la fois morphologique et dynamique des voies aériennes supérieures. Son intérêt peut être diagnostique ou thérapeutique. C’est un geste chirurgical court, d’environ 20 minutes, dont la stimulation douloureuse est intense mais brève, ne laissant que des douleurs postopératoires modérées [1]. Les patients bénéficiant d’une LDS représentent souvent des terrains particuliers. Il s’agit surtout de patients alcoolo-tabagique et qui ont plusieurs pathologies associés, telles une maladie athéromateuse diffuse, une BPCO, un cancer bronchique, une cirrhose, une dénutrition [1]. La lésion à explorer peut avoir des répercussions sur la possibilité de ventilation au masque ou d’intubation. Ainsi l’évaluation pré-anesthésique doit évaluer l’importance de l’intoxication alcoolo-tabagique et son retentissement sur la fonction respiratoire et hépatique et également le degré d’obstruction des voies aériennes supérieurs (VAS). Au terme de cette évaluation, la conduite anesthésique et la gestion des VAS va dépendre du siège et de la taille de la lésion à explorer. La LDS propose une excellente exploration laryngoscopique du larynx, mais elle est inévitablement sujette de plusieurs problèmes anesthésiques dont la gestion actuelle reste délicate. C’est un geste réflexogène, irritant et court. Ce temps opératoire court exige une induction, un maintien et un rétablissement rapide de l’anesthésie [3]. La profondeur de l’anesthésie doit être suffisante pour limiter les répercussions cardiovasculaires et modulables pour permettre un approfondissement rapide et une bonne réversibilité [1].

Plusieurs techniques anesthésiques peuvent être proposées afin de résoudre ce défi, ces techniques dépendent de la taille de la lésion et de son siège qui conditionne le matériel a utilisé ainsi que la technique de ventilation. En cas de pathologie glottique, la sonde d’intubation gène l’endoscopie et le traitement d’une lésion surtout si postérieur d’où l’intérêt de technique avec ventilation spontanée avec sédation ou même avec anesthésie locale de la base de la langue et du larynx. Ces technique peut courir au risque d’apnée si la sédation est très profonde ou dans le cas contraire à un spasme si la sédation est légère ainsi que le risque d’inhalation post opératoire par persistance d’anesthésique locale d’où sa limitation [4]. La technique de choix est représentée par le jet ventilation sur un fin cathéter [4] positionnée soit en tran-trachéal après ponction inter-crico-thyroidienne, soit en trans-glottique en cas de contre-indications à la ponction. La ventilation spontanée et le jet ventilation intercricothyroidienne ont l’avantage de laisser le champ complètement libre au chirurgien et d’éviter de générer avant l’endoscopie un saignement dû à des tentatives d’intubation parfois difficile [1]. En cas de pathologie sus ou sous glottiques une anesthésie générale avec intubation peut être réalisée, la sonde d’intubation doit être de petit calibre [5], la présence de cette sonde diminue le confort chirurgical, mais offre la possibilité d’une anesthésie par halogénés [1]. L’induction peut être intraveineuse par propofol dont les propriétés pharmacocinétiques et pharmacologiques sont intéressantes par rapport au thiopental et à l’étomidate [1], ou inhalatoire par le sevoflurane ainsi qu’un morphiniques de courte durée d’action a fin d’atténué l’hypertension artérielle et la tachycardie voire l’hypertension artérielle pulmonaire induite par l’endoscopie [1]. Le curare n’est pas systématique à l’induction [6], une intubation sans relaxation musculaire a été développé chez les adultes mais également en pédiatrie grâce à une utilisation combinée de propofol et de rémifantanil dont l’intérêt a été démontré chez les patients programmés qui doivent bénéficier de geste bref ce qui offre des conditions favorables pour l’intubation ainsi qu’aux procédures laryngoscopiques [3].

L’utilisation de curares reste limitée chez les patients qui subissent une laryngoscopie en suspension en raison de la durée limitée du geste et le risque de la curarisation résiduelle et par conséquent le cout total de la procédure [3]. Cependant le curare garde son intérêt dans la relaxation musculaire en anesthésie générale facilitant ainsi l’intubation mais également la technique chirurgicale, le maintien d’une relaxation musculaire durant l’intervention laryngée [3], et surtout il permet d’assurer une immobilité lors de geste de haute précision (laser, microchirurgie) ou pendant les temps endoscopiques à haut risque de perforation [1]. La succinylcholine possède comme avantages une durée d’action courte et une action plus rapide et plus intense sur les muscles laryngés que sur les autres muscles. Ses effets indésirables en limitent l’utilisation [1]. Le rocuronium, appartenant à la classe des curares stéroïdiens, s’est révélé comme alternative intéressante à la succinylcholine. Il a une action rapide, un effet moins allergisant et surtout ces contre-indications sont moins nombreux. Mais sa durée d’action prolongée reste un handicap à son utilisation dans les actes opératoires de brève durée. La néostigmine est peu utilisée, cela est dû à ses contre-indications fréquentes, son pic d’action relativement lent, la nécessité d’administrer simultanément un anti-cholinergique et l’impossibilité de neutraliser un bloc neuromusculaire profond [7]. Ceci la limite lors du LDS vue la rapidité du geste.

Les effets indésirables de la néostigmine résultent d’une action hors la jonction neuromusculaire. Une co-administration avec un anticholinergiques [8] tel que l’atropine et le glycopyronium permet de neutraliser ces effets secondaires, bien que ces médicaments peuvent être à l’origine d’autres effets secondaires qui leurs sont propres à savoir une tachycardie, une diminution des sécrétions et une vision floue [9]. L’avènement du sugammadex a relancé l’intérêt des curares stéroïdiens. Il a été approuvé comme le premier agent ciblé pour décurariser un bloc neuromusculaire profond induit par le rocuronium ou le vecuronium avec un meilleur profil sécuritaire [8]. Il encapsule les composés stéroïdiens, et cela sans avoir d’effets cholinergiques [9]. Il permet d’antagoniser le rocuronium en cas d’intubation impossible et également d’éviter au maximum le risque de curarisations résiduelle et ainsi une meilleur récupération clinique [8] avec une récupération neuromusculaire complète obtenue en moins de 3 minutes, quelle que soit la profondeur du bloc neuromusculaire. Ses avantages par rapport aux anticholinergiques ont été démontrés dans de nombreuses études [8]. Comparé à la néostigmine, la récupération postopératoire des facultés nociceptives et physiologiques était plus précoce avec le sugammadex, ainsi l’utilisation du sugammadex a permis d’améliorer les perspectives chirurgicales sur les activités quotidiennes avec un plus haut niveau de satisfaction [8]. La même étude, a objectivé que le temps moyen de récupération du bloc neuromusculaire avec un TOF à 0,9 était de 3 min pour le sugammadex contre 9 min pour la néostigmine [8]. Le sugammadex peut donc permettre une inversion rapide d’un bloc neuromusculaire profond [9] permettant ainsi de réduire la morbidité postopératoire sans effets indésirables connues et par conséquent réduire le cout de la procédure [10] en assurant une sécurité pour le patient et un confort pour l’anesthésiste.

## Conclusion

La laryngoscopie directe en suspension nécessite à la fois un contrôle permanent du niveau d’anesthésie et un réveil rapide [**1**]. L’association du rocuronium et sugammadex semble un algorithme envisageable. Il permet une facilité du geste chirurgicale en offrant une bonne qualité de curarisation, une meilleure relaxation musculaire, une immobilité lors des gestes de haute précision et une meilleure sécurité respiratoire [**7**].
